# Corticospinal Excitability of Trunk Muscles during Different Postural Tasks

**DOI:** 10.1371/journal.pone.0147650

**Published:** 2016-01-25

**Authors:** Shin-Yi Chiou, Sam E. A. Gottardi, Paul W. Hodges, Paul H. Strutton

**Affiliations:** 1 The Nick Davey Laboratory, Human Performance Group, Division of Surgery, Department of Surgery and Cancer, Faculty of Medicine, Imperial College London, Charing Cross Hospital, London, United Kingdom; 2 The University of Queensland, NHMRC Centre of Clinical Research Excellence in Spinal Pain, Injury and Health, School of Health and Rehabilitation Science, Brisbane, Queensland, Australia; Scientific Institute Foundation Santa Lucia, ITALY

## Abstract

Evidence suggests that the primary motor cortex (M1) is involved in both voluntary, goal-directed movements and in postural control. Trunk muscles are involved in both tasks, however, the extent to which M1 controls these muscles in trunk flexion/extension (voluntary movement) and in rapid shoulder flexion (postural control) remains unclear. The purpose of this study was to investigate this question by examining excitability of corticospinal inputs to trunk muscles during voluntary and postural tasks. Twenty healthy adults participated. Transcranial magnetic stimulation was delivered to the M1 to examine motor evoked potentials (MEPs) in the trunk muscles (erector spinae (ES) and rectus abdominis (RA)) during dynamic shoulder flexion (DSF), static shoulder flexion (SSF), and static trunk extension (STE). The level of background muscle activity in the ES muscles was matched across tasks. MEP amplitudes in ES were significantly larger in DSF than in SSF or in STE; however, this was not observed for RA. Further, there were no differences in levels of muscle activity in RA between tasks. Our findings reveal that corticospinal excitability of the ES muscles appears greater during dynamic anticipatory posture-related adjustments than during static tasks requiring postural (SSF) and goal-directed voluntary (STE) activity. These results suggest that task-oriented rehabilitation of trunk muscles should be considered for optimal transfer of therapeutic effect to function.

## Introduction

Trunk muscles play an essential role in the maintenance of upright posture and this has been extensively studied during limb movements, support surface translations and perturbations applied to the trunk [1–4]. These muscles are also activated for voluntary goal-directed movements such as simple movements of the trunk into flexion, extension and rotation [5–7]. It is generally accepted that the motor cortex is involved in voluntary tasks [8], with relatively greater contributions from subcortical structures in postural tasks [9]. In leg muscles, studies using transcranial magnetic stimulation (TMS) have shown motor cortical involvement in both postural and voluntary tasks [10, 11], but cortico-muscular coherence, between EEG recorded over the motor cortex and electromyographic (EMG) activity is lower in postural than voluntary goal-directed tasks [12, 13]. This implies different neural mechanisms may mediate control of the two distinct classes of motor function.

Postural functions of the trunk muscles are diverse and are controlled by brain stem (e.g. vestibular [14], reticular [15]), spinal (e.g. stretch reflexes [16, 17]) and cortical [9] mechanisms in humans and animals. In these cases, cortical activation might be expected to be less than for voluntary tasks, but not absent [9]. An array of non-cortical inputs to the motoneuron pool of the paraspinal muscles has been described in animals [15]. Some postural adjustments are considered to have greater contribution from the motor cortex. In particular, this is thought to be the case for anticipatory postural adjustments (APAs), which are initiated within a time frame (-100 to +50ms [18]) that either precedes movement or is too fast to be a result of a reaction to afferent input from the periphery [18–20] and thus preplanned (i.e. before any somatosensory feedback) by the nervous system. For instance, voluntary limb movements are accompanied by activity of muscles of the other limbs [21, 22] and trunk [4, 19, 23] in advance of the movement in a manner that is specific to counteract the reactive forces from the movement. These adjustments are considered to be controlled by cortical mechanisms as animal and human studies show abnormal APAs in the presence of lesions of the cerebral cortex [24–26], and both forelimb movement and the associated APAs are initiated by stimulation of cells of the motor cortex in cats [27]. In humans, excitability of corticospinal projections to leg muscles involved in step initiation is increased in the time-window of the APA when probed with TMS [28]. Although the cortical regions involved in programming APAs include the supplementary and primary motor cortices [29], their contribution to control of trunk muscles during APAs has not been investigated.

Corticospinal projections make a major contribution to limb muscles during voluntary movements [30, 31], particularly for fine dextrous control of distal segments [32]. Although the cortical representation of the trunk on the motor homunculus is small relative to the hand [33, 34], TMS studies confirm corticospinal projections to trunk muscles [35–38], but the involvement of corticospinal inputs in voluntary goal-directed activation of trunk muscles has received little attention. One study assessed excitability of corticospinal inputs to the erector spinae (ES) muscles in a static postural task (sustained abduction of the contralateral arm) [39]. The amplitude of the motor evoked potentials (MEP) from TMS increased during this task. However, MEP amplitude increases when excitability of the cortex or motoneurone is increased [40], and as MEP amplitude co-modulated with EMG amplitude (i.e. motoneuron excitability), data from that study cannot provide information regarding the cortical involvement.

This study was designed to use TMS to investigate the changes in corticospinal excitability to the trunk muscles during voluntary and postural tasks. We hypothesised that ES muscle activity during a voluntary trunk extension task (static trunk extension; STE) would involve greater input from the motor cortex than ES muscle activity associated with the simple static postural challenge to keep the spine/body upright when holding the arms in front of the body in a sustained manner (static shoulder flexion; SSF). Further, we hypothesised that any facilitation of the MEP would be similar for the voluntary STE task and when the ES are activated as a component of the APA accompanying dynamic shoulder flexion (DSF). This study aimed to test these hypotheses in healthy young adults.

## Materials and Methods

### Participants

Twenty healthy adults (male: female 10:10; mean (SD) age 22 (3) years, height 174 (9) cm, body mass 70 (11) kg) were recruited from students and staff at the corresponding author’s institution. Participants were excluded if they had a history of musculoskeletal abnormalities of the upper extremity, back musculature and axial skeleton (e.g. scoliosis and low back pain); or met the criteria for exclusion for the use of TMS (i.e. metal implants, cardiac pacemaker, history of epilepsy or fits, previous brain injury, neurosurgery, neurological disorders, psychological disorders, actively taking antidepressant or other neuromodulatory drugs [41]. The institutional Medical Research Ethics Committee approved the study and all participants provided written informed consent.

### Electromyography (EMG)

Bilateral EMG recordings were obtained from erector spinae (ES) at the 4^th^ lumbar vertebral level (L4), rectus abdominis (RA) and deltoid. Pairs of Ag/AgCl electrodes (self-adhesive, 2 cm diameter, CareFusion, UK) were positioned approximately parallel to the muscle fibre orientation. A ground electrode was placed over the left anterior superior iliac spine. For ES, electrodes were positioned 3 cm either side of the spinous processes with an inter-electrode distance of 2 cm; for RA, 3 cm lateral to the midline immediately below the level of the umbilicus; and for deltoid, over the anterior muscle belly with 3 cm separation between the electrodes. EMG data were filtered (10–1000 Hz), amplified (1000×; Iso-DAM, World Precision Instruments, UK) and sampled at 2 kHz using a Power 1401 data acquisition system and Signal v5 software (Cambridge Electronic Design [CED], UK) connected to a personal computer for subsequent offline analysis.

### Transcranial magnetic stimulation (TMS)

**Fig 1:**
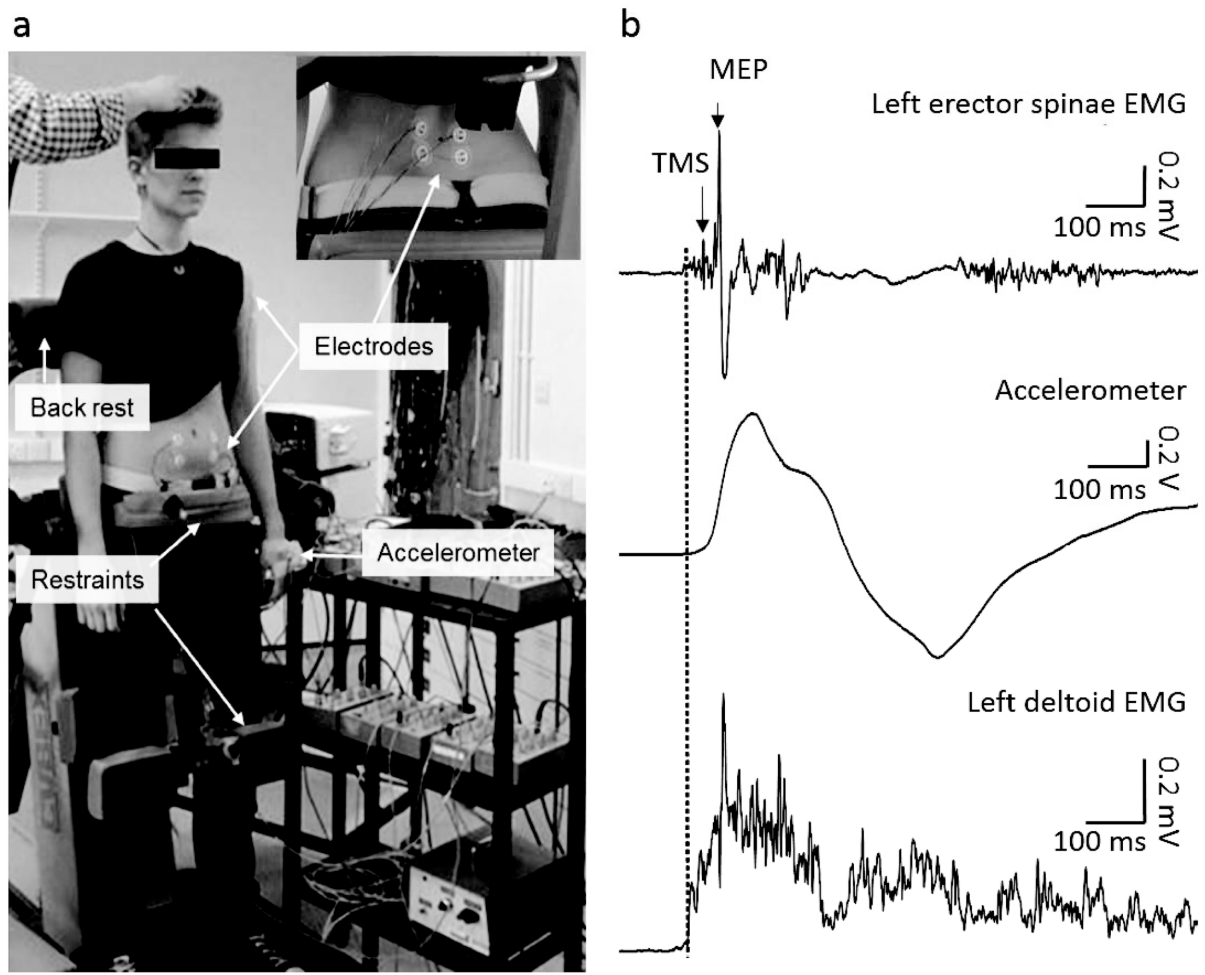
Experimental setup. (a) Participants stood upright on a restraining device with pelvis and knees securely fixed to minimise movement of pelvis and lower limbs. Electrodes were attached over erector spinae at the 4^th^ lumbar vertebral level (panel in top right), rectus abdominis and left deltoid. An accelerometer was positioned on the dorsum of the hand contralateral to the stimulation. (b) Representative data from a single subject showing left erector spinae EMG, left deltoid EMG and accelerometer data during dynamic shoulder flexion task. TMS was delivered 25 ms after the onset of deltoid EMG (dotted vertical line).

TMS was delivered to the motor cortex using a Magstim 200^2^ mono-phasic stimulator (The Magstim Company Ltd., UK) connected to a figure-of-eight coil (wing outer diameter 10 cm), positioned over the approximate location of primary motor cortex at a site which elicited a maximal motor evoked potential (MEP) in the contralateral ES muscle. The position of the coil was marked on the scalp to ensure consistent placement of the coil throughout the experiments. The coil was orientated 45° relative to the midline with the handle pointing posteriorly to induce a current flow in the anteromedial direction. Participants stood upright with their pelvis and knees strapped securely to minimise movement of pelvis and lower limbs (Fig 1a). Active motor threshold (AMT) of the ES muscle was established while participants performed low level voluntary isometric back extension. Threshold was defined as the lowest intensity of TMS that evoked visible MEPs in at least three of six consecutive trials. The procedure was repeated to establish AMT for the other hemisphere. As 10 out of 20 participants had a difference in AMTs between the two hemispheres of greater than 10%, the TMS was applied to the more excitable hemisphere, i.e. the one with the lower AMT [42]. The intensity of TMS for the main experiment was set to 1.2xAMT.

### Experimental procedure

Participants performed three brief (~2 s) maximum voluntary contractions (MVC) of trunk extension against the back-rest of the restraining device, with at least 10 s rest between contractions; strong verbal encouragement was provided throughout. During each MVC contraction, a light-box displaying EMG activity of ES was adjusted to show all 10 lights; this was used subsequently to allow subjects to maintain consistent levels of ES contraction during the SSF and STE tasks.

MEPs were evoked by TMS while participants performed three tasks: bilateral dynamic shoulder flexion (DSF), bilateral static shoulder flexion (SSF), and static trunk extension (STE). In the DSF task, participants were instructed to perform bilateral shoulder flexion by raising both arms to 90° as fast as possible without flexing the elbow or wrist in response to the verbal cue, ‘go’, from the experimenter. The rise in amplitude of the deltoid EMG was detected using the threshold-crossing feature of Signal and this triggered the delivery of the TMS pulse with a 25-ms delay. This timing ensured that the TMS pulse was applied during the anticipatory postural activation of the ES muscle, before any feedback from arm movement or perturbation to the spine from the arm movement could modify the ES muscle activation (i.e. ~50ms [43]). A custom built accelerometer was positioned on the dorsum of the hand contralateral to the stimulated brain hemisphere to record the time of start and the end of the shoulder flexion (Fig 1b). In the SSF task, participants held their shoulders flexed to 90° against an elastic physiotherapy band secured around their wrists and attached inferiorly to a stable fixation point. In the STE task, participants performed isometric trunk extension against the back-rest. For the SSF and STE tasks, TMS pulses were delivered at random intervals (>5 s apart) during the tasks. The pre-stimulus ES EMG amplitude was measured during the DSF task (see data analysis below) and this level of EMG activity was used to match the ES activity during the SSF and STE tasks using the light-box as feedback. The DSF task was therefore the first task performed in each trial and followed by either SSF or STE tasks, which were performed in a random order. Each of the trials in the static tasks was performed as a separate short (~1s) contraction. Subjects contracted to try to match the required level of ES EMG activity prior to the TMS being delivered and were instructed to relax after the TMS. Participants repeated each task until 10 trials were obtained in which the pre-stimulus EMG activity was matched (mean [±SD] number of trials performed: SSF: 11.65±2.13; STE: 11.1±2.22); a rest period of at least 5 s was given between individual contractions to avoid fatigue.

### Data analysis

EMG data from the 10 trials were averaged and vertical cursors positioned at the start and finish of the MEP. The average MEP peak-to-peak amplitudes for each task were measured from ES and RA EMG recordings. The MEP latency for each muscle was identified from the average rectified EMG traces and was defined as the time at which the EMG amplitude exceeded 2 SD above the mean pre-stimulus EMG level. Pre-stimulus EMG obtained during the tasks was calculated for ES and RA muscles as the root-mean square amplitude (rmsEMG) in a 25-ms window and 150-ms window prior to the stimulus in the DSF and SSF/STE tasks, respectively. The rmsEMG in a 10-ms window immediately after the TMS pulse in the DSF task was also calculated to examine if there was a significantly higher level of EMG when the volleys evoked by the TMS would likely be arriving at the spinal motoneurons [44]. Pre-stimulus rmsEMG of deltoid obtained during the DSF and SSF tasks was calculated over a 25-ms window prior to the stimulus.

### Statistical analysis

Data were analysed using Statistical Program for the Social Sciences (SPSS) version 21 (IBM Corp, Armonk, NY). Repeated-measures ANOVA with paired t-tests as post-hoc tests were used to determine whether MEP amplitudes, MEP latencies and pre-stimulus rmsEMG differed between TASKs (DSF, SSF and STE). Paired t-tests were also used to examine changes in ES rmsEMG between the 2 time windows (10ms following TMS and 25ms prior to TMS) and differences in deltoid rmsEMG amplitude between the DSF and SSF tasks. Friedman Test was used to compare the RA MEP amplitudes, as MEPs were not observable for all participants. Statistical significance was set at p<0.05 and Bonferroni correction was applied to adjust for multiple comparisons. Data are presented as mean±SD in the text and as mean±SEM in the figures.

## Results

The mean threshold to elicit a MEP from ES with stimulation to the less excitable hemisphere (i.e. hemisphere with the higher AMT) was 67.4%, (range: 50–85%) of maximum stimulator output (%MSO); mean AMT for the more excitable hemispheres was 58.8%MSO (range: 45–85%MSO). The ES rmsEMG activity during a 0.5-s window during the MVCs was not different between sides (t(19) = -0.88, *p = 0*.*39*) with a mean amplitude of 0.24±0.13 mV. In the DSF task, the mean rmsEMG amplitude in ES during the 25-ms window prior to the TMS pulse (0.06±0.04 mV) was not different than that during the 10-ms window following the TMS pulse (0.08±0.05 mV; t(14) = -1.59, *p* = 0.14). Pre-stimulus deltoid rmsEMG amplitude was not different between DSF (0.31±0.11 mV) and SSF tasks (0.32±0.15 mV; t(19) = -0.31, *p* = 0.76). The average velocity of shoulder movement was 102(±21)°s^-1^.

**Fig 2:**
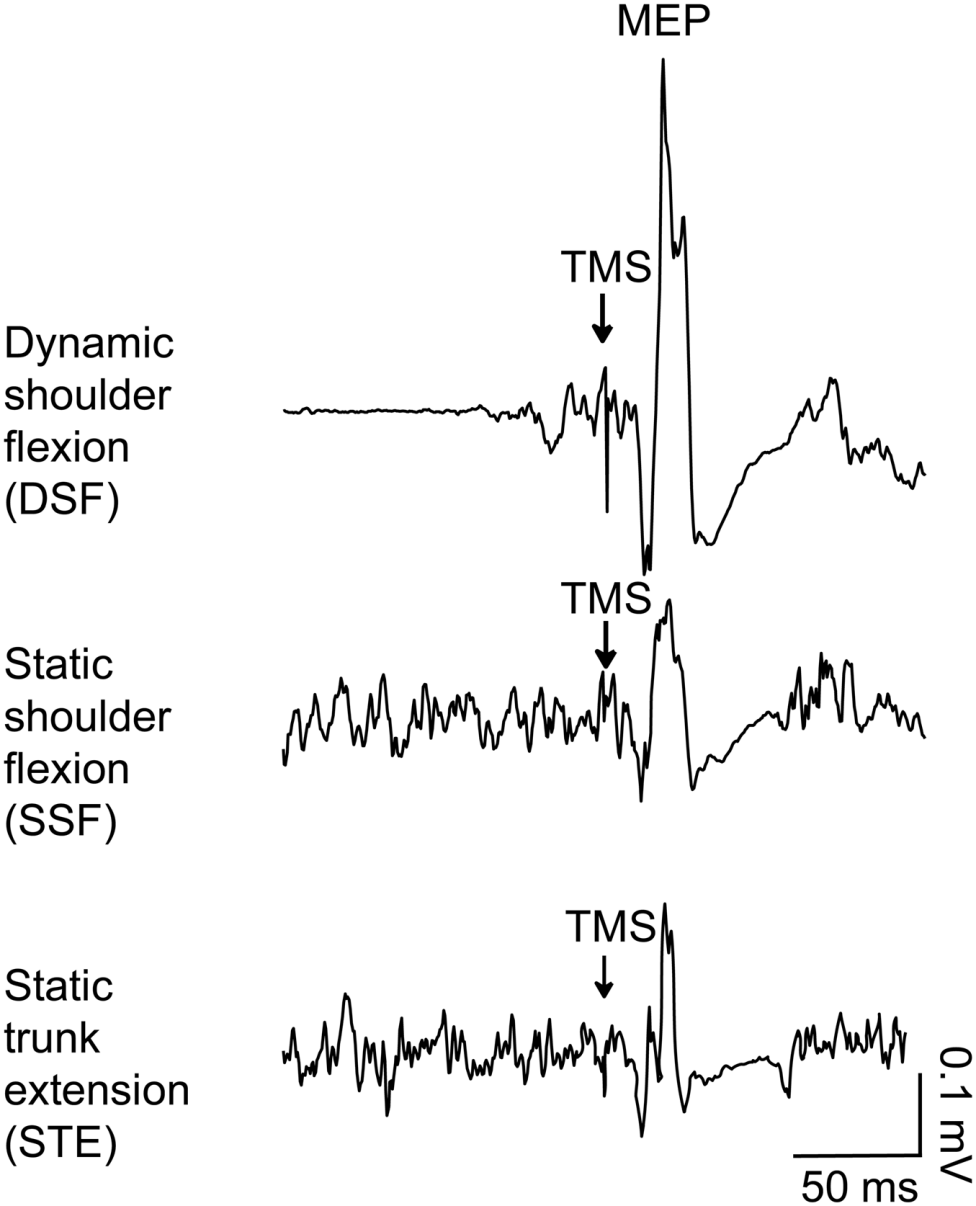
Averaged data (10 stimuli) from a representative subject showing the motor evoked potentials (MEP) in erector spinae during the different tasks. Arrows indicate the time of transcranial magnetic stimulation (TMS) over the motor cortex. There is a clear increase in EMG prior to the TMS in the bilateral dynamic shoulder flexion (DSF). Tonic EMG activity is present in the two static tasks; bilateral static shoulder flexion (SSF) and static trunk extension (STE).

**Fig 3:**
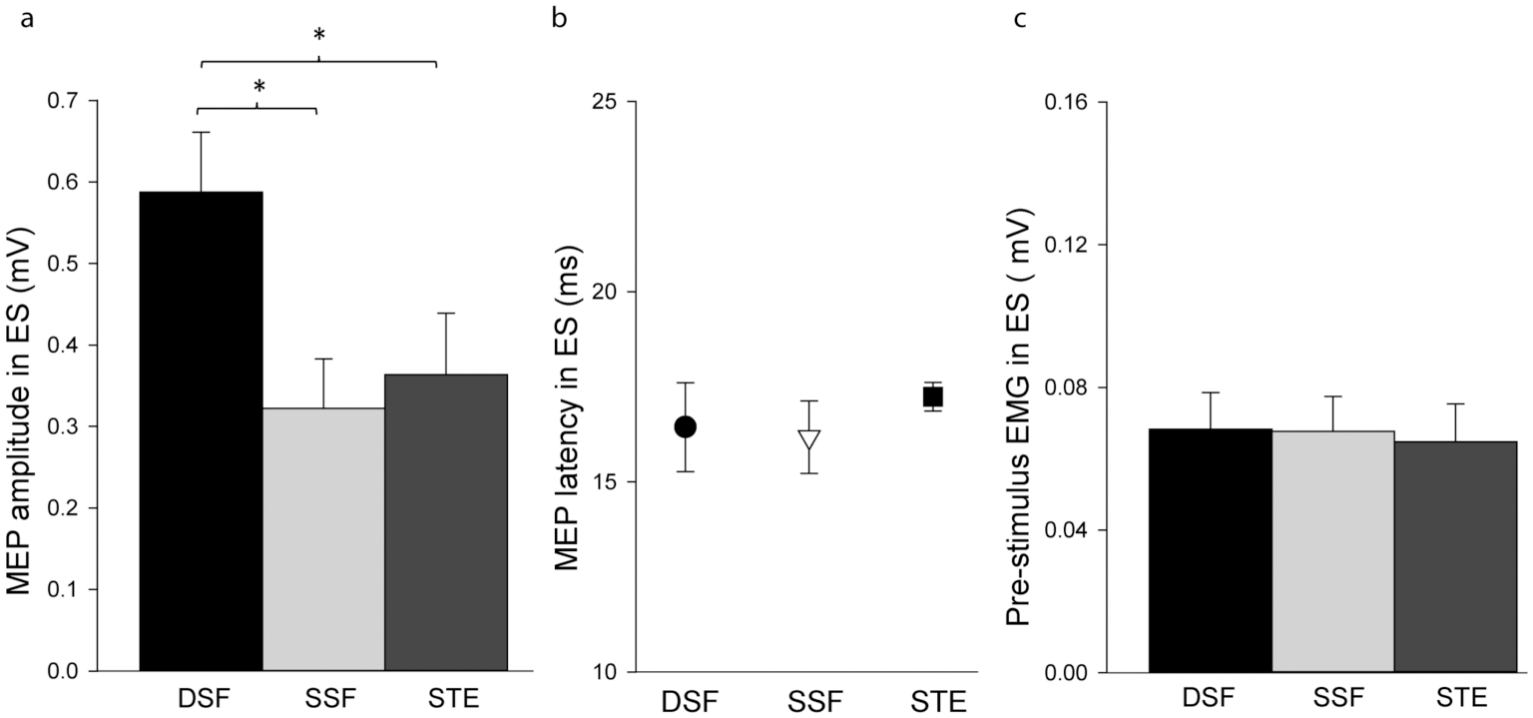
Group mean (SEM) data of motor evoked potentials (MEPs) and EMG recorded from erector spinae (ES). (a) Amplitudes of MEPs obtained during the bilateral dynamic shoulder flexion (DSF), bilateral static shoulder flexion (SSF) and static trunk extension (STE) tasks. (b) MEP latencies in ES in the three tasks. (c) Pre-stimulus ES rmsEMG amplitude during the three tasks. *—significant difference, p<0.017.

Fig 2 shows the averaged (10 frames) EMG traces of the ES during the three tasks from a representative participant. The ES MEP amplitude differed between TASKs (F_2,38_ = 15.94, *p*<0.001). Post-hoc tests showed that the MEP amplitude in the DSF task (0.59±0.33 mV) was greater than the SSF (0.32±0.27 mV; *p*<0.001) and STE tasks (0.36±0.34 mV; *p* = 0.002; Fig 3a). There was no significant difference between the SSF and STE tasks (*p* = 0.33; Fig 3a). There was no effect of TASK (F_1.45, 26.06_ = 0.45, *p* = 0.58) on MEP latencies (DSF: 16.43±5.10 ms; SSF: 16.17±4.17 ms; STE: 17.24±1.64 ms; Fig 3b). Consistent with our objective to match ES EMG prior to the TMS, pre-stimulus ES EMG did not differ between TASKs (DSF: 0.06±0.04 mV; SSF: 0.06±0.04 mV; STE: 0.06±0.04 mV; F_2,38_ = 0.88, *p* = 0.42; Fig 3c).

**Fig 4:**
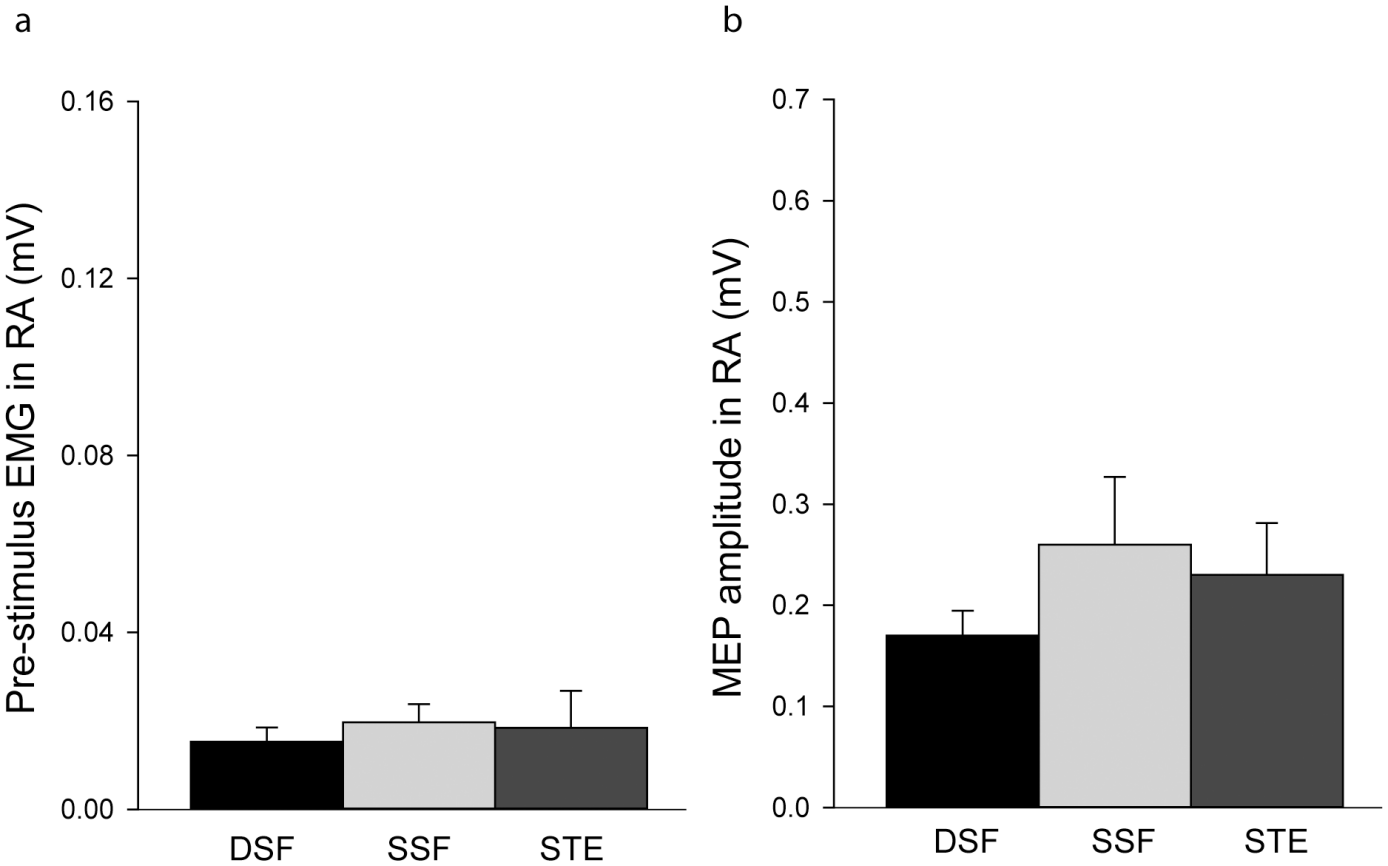
Group mean (SEM) data of motor evoked potentials (MEPs) and EMG recorded from rectus abdominis (RA). (a) Amplitudes of MEPs obtained during the bilateral dynamic shoulder flexion (DSF), bilateral static shoulder flexion (SSF) and static trunk extension (STE) tasks. (b) Pre-stimulus RA rmsEMG amplitude during the three tasks.

RA MEPs were less consistently elicited than those of ES. For the DSF task, 15 subjects showed consistent MEPs in RA, 9 in SSF and 9 in STE. There was no effect of TASK (χ2(6) = 0.33, *p* = 0.85) on the MEP amplitudes (DSF: 0.17±0.11 mV; SSF:0.26±0.30 mV; STE:0.23±0.23 mV; Fig 4a) or pre-stimulus EMG activity in RA (DSF: 0.01±0.01 mV; SSF: 0.02±0.01 mV; STE: 0.02±0.03 mV; F_1.13, 21.41_ = 1.03, *p* = 0.42; Fig 4b).

## Discussion

The results of this study show that corticospinal excitability differs between modes of activation of the ES muscles, when the amplitude of ES EMG (i.e. motoneuron excitability) was controlled across tasks. As expected, corticospinal excitability in the ES was greater during the APA (DSF), which we hypothesised would involve a greater cortical contribution, than the sustained postural activation (SSF), which we hypothesised would involve lesser relative contribution from the motor cortex. However, contrary to our hypothesis, corticospinal excitability in the ES during the voluntary goal-directed task (STE) was less than that for the APA (DSF) (which we predicted would be similar) and no different to the SSF task (which we predicted would be less than STE). These observations challenge the contemporary understanding of neural control of trunk muscles.

### Corticospinal involvement in APAs of trunk muscles

Rapid movement of the upper limbs requires complex postural adjustments of the trunk (whether anticipatory or compensatory) to counteract the effect of the imposed forced on centre of mass and the orientation of the spine [1, 45, 46]. Our observation of greater ES MEP amplitude during the APA period (DSF) than that during the sustained postural task (SSF), despite similar pre-stimulus EMG amplitude, concurs with the greater involvement of the motor cortex in this task, and is consistent with findings of previous studies of trunk and limb muscles [19, 47, 48], and imaging findings of cortex activation during APAs [29]. Shoulder flexion involves anticipatory activity of ES, and not RA [4, 18], thus the lack of difference in RA MEP amplitude between the tasks implies the enhancement of corticospinal excitability was specific to the muscles involved in the task. One caveat is that the location for stimulation of RA was not optimised. However, changes in corticospinal excitability induced by experimental pain have been observed in surrounding muscles not targeted by the coil location [49] and we believe that any excitability changes would have been observed if present in inputs to RA. Whether the pattern of corticospinal excitability observed for ES (higher during DSF than SSF) would be observed for RA in an upper limb task in an alternative direction (e.g. shoulder extension), remains to be determined, but is likely given the distinct patterns of activity observed with shoulder movements in differing directions [4].

Data from patients with low back pain further supports the role of the motor cortex in control of trunk muscles as a component of APAs. Individuals with LBP exhibit changes in excitability of corticospinal inputs to the trunk muscles [42, 47, 50–52], delayed components of APAs [53] and a correlation between the corticospinal changes and the delays in components of the APA [47, 48].

An unexpected finding of this study was that of greater corticospinal excitability during the APA than during a voluntary goal directed task. Task-dependency of motor cortical excitability has been widely observed in limb muscles [54–56], and has been shown for trunk muscles, albeit during voluntary trunk contractions; corticospinal excitability of trunk muscles during voluntary forced expiratory efforts is higher than during bilateral voluntary trunk extension or flexion [57, 58], despite similar levels of background EMG. In that study it is unclear why the cortical contribution during a voluntary trunk movement task is less than during a respiratory-related task, which (like postural activity) is thought to involve greater contribution from subcortical structures [59]. Further, a range of brain regions are involved in postural control; for example, an imaging study showed enhanced activation in the posterior parietal cortex and supplementary motor area preceding an external perturbation when a warning cue was provided [60]. Cognitive-motor processes are also suggested since dual-task experiments, in which subjects simultaneously perform a postural task and a cognitive task, demonstrated alterations in the performance of the postural task [61–63]. As M1 has structural connections with other brain regions [64], its excitability is likely reflective of projections from these regions involved in the task. Taken together these data imply that simple voluntary trunk extension involves less cortical activation than postural tasks requiring APAs.

Our method does not provide an absolute measure of corticospinal involvement in the generation of ES activity, but instead a relative measure between tasks. Thus, motor cortical involvement is likely in both task types, but greater in the APA than in the sustained voluntary contractions. There are several possible explanations for the difference between tasks. First, this might be related to the dynamic nature of the DSF task, MEPs have been shown to be larger during dynamic versus static contractions in muscles which are the prime-movers of the task [65, 66], whether this applies to the non-prime movers (i.e. the trunk muscles in the DSF task) is unknown. Contribution of cortical inputs might be greater during the dynamic component of the task when EMG activity undergoes change (start of movement or change in force), and may be limited for the sustained component of the voluntary task. However, our data showed that the EMG activity in ES during the DSF task did not increase significantly between the pre-stimulus period (25 ms prior to the TMS) and the post-stimulus period (10 ms after the TMS), when the TMS-evoked volleys are likely to be arriving at the spinal motoneurons [44]. It should be noted that although the experimental setup likely increased body stability which has been shown to decrease APAs [67, 68], the increased excitability observed during the DSF might be further increased in a setup in which there is no restraint. Second, cortical excitability undergoes a major change in advance of movement [69, 70] whereas alternative sources of neural input from subcortical regions, such as the basal ganglia [71], exhibit relatively greater activity during sustained than dynamic elements of movement. In contrast to this proposal, it has been shown that relationship between oscillations in cortical and muscle activity (which should reflect cortical contribution to a task) is diminished during the ramp (dynamic) compared to the hold (static) phase [72]. Further work is required to directly compare corticospinal excitability during dynamic and static voluntary efforts.

Second, voluntary control of trunk muscles may involve less contribution from corticospinal inputs than limb muscles [15]. Other sources of input could include basal ganglia, brain stem, reticulospinal and other areas, from which the paraspinal muscles might receive a greater contribution of drive than limb muscles [15], or from spinal circuits such as muscle spindle inputs and propriospinal pathways [17]. Third, it has been proposed that trunk muscles receive greater input from one hemisphere; analysis of data from ipsilateral and contralateral inputs has revealed one cortex with a lower threshold for both ipsilateral and contralateral projections [47]. This was interpreted to suggest that, unlike primarily contralateral cortical control of voluntary limb actions, bilateral goal-directed trunk muscle contractions might receive primary drive from a single hemisphere. Thus, greater MEP facilitation would only be detected if we had stimulated the “driving” hemisphere, and if this was variable between participants we will have underestimated the facilitation of the MEP during the voluntary action. Although we elected to stimulate the hemisphere with the lowest threshold for contralateral MEPs, the study of Tsao et al., (2008) implies that the cortex with the lowest threshold for ipsilateral MEPs may be the more important consideration for determination of the “driving” hemisphere [47] and this is not necessarily the same side as the more excitabile hemisphere for contralateral MEPs.

### Similar corticospinal involvement in voluntary (STE) and sustained postural activation (SSF) of ES

We did not expect corticospinal excitability to be similar for the STE and SSF tasks. The tonic postural ES activity associated with the anterior gravitational load on the trunk from sustained shoulder flexion in the present study was expected to involve less contribution from the motor cortex than voluntary goal-directed activation of the muscle during the STE task. Consistent with this argument, there is less coherence between cortical activity and activation of soleus in standing [12], and voluntary attempts to match activity of soleus in a voluntary effort to that observed in standing require substantial effort in excess of that in the postural task, implying greater subcortical contribution to drive in the postural task [73]. Numerous subcortical regions (e.g. vestibular, basal ganglia, etc.) also contribute to maintenance of on-going postural tone [9, 15], which may result in overall MEP facilitation during the static postural task. Alternatively, given that activation of ES was coupled to the voluntary maintenance of arm position in the present paradigm, this may have maintained a greater drive from the cortex related to the deltoid activation. The potential impact of subtle differences in postural task characteristics on cortical contribution to control requires further investigation.

### Clinical relevance

The current findings have several potential implications for rehabilitation of disorders involving activation and control of trunk muscles. First, the data point to task specificity in the involvement of cortical and subcortical inputs to trunk muscles, in a manner that is not completely predictable based on the “nature” of the task. This highlights the need for task-oriented rehabilitation of trunk muscles for optimal transfer to function.

### Conclusion

In conclusion, the current results show that the involvement of motor cortical pathways driving trunk muscles differs between tasks. The novel observation of this study is that this is not simply predictable based on the nature of the task and the current understanding of control of limb muscles during voluntary tasks versus those of a more “automatic” nature. Additional work is required to understand the differences in fundamental principles that drive neural control of limb and trunk muscles.
